# Whole genome re-sequencing identifies a mutation in an ABC transporter (*mdr2*) in a *Plasmodium chabaudi* clone with altered susceptibility to antifolate drugs^☆^

**DOI:** 10.1016/j.ijpara.2010.08.008

**Published:** 2011-02

**Authors:** Axel Martinelli, Gisela Henriques, Pedro Cravo, Paul Hunt

**Affiliations:** a Centro de Malaria e Outras Doenças Tropicais/IHMT/UEI Biologia Molecular, Universidade Nova de Lisboa, Rua da Junqueira 96, 1349-008 Lisbon, Portugal; b Institute for Immunology and Infection Research, School of Biological Sciences, University of Edinburgh, Edinburgh, UK; c Centre for Immunity, Infection and Evolution, School of Biological Sciences, University of Edinburgh, Edinburgh, UK

**Keywords:** *Plasmodium*, Sulphadoxine, Folate, Mutation, *mdr2*

## Abstract

In malaria parasites, mutations in two genes of folate biosynthesis encoding dihydrofolate reductase (*dhfr*) and dihydropteroate synthase (*dhps*) modify responses to antifolate therapies which target these enzymes. However, the involvement of other genes which modify the availability of exogenous folate, for example, has been proposed. Here, we used short-read whole-genome re-sequencing to determine the mutations in a clone of the rodent malaria parasite, *Plasmodium chabaudi*, which has altered susceptibility to both sulphadoxine and pyrimethamine. This clone bears a previously identified S106N mutation in *dhfr* and no mutation in *dhps*. Instead, three additional point mutations in genes on chromosomes 2, 13 and 14 were identified. The mutated gene on chromosome 13 (*mdr2* K392Q) encodes an ABC transporter. Because Quantitative Trait Locus analysis previously indicated an association of genetic markers on chromosome 13 with responses to individual and combined antifolates, MDR2 is proposed to modulate antifolate responses, possibly mediated by the transport of folate intermediates.

## Graphical abstract

**Figure:**
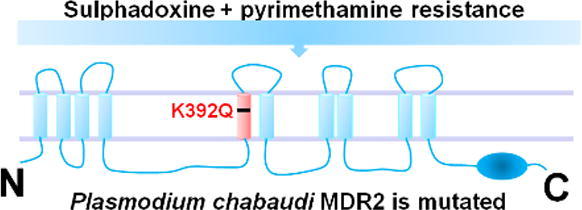

**Research highlights:**

► A pyrimethamine+sulphadoxine-resistant rodent malaria genome was sequenced. ► Its genome sequence was compared with that of a sensitive progenitor. ► Four point mutations were identified: on chromosomes 2, 7, 13 and 14. ► The K392Q mutation in *mdr2* is genetically linked to sulphadoxine resistance. ► *mdr2* is a novel candidate gene underlying antifolate resistance in malaria.

## 1 Introduction

**Supplementary Fig. 1:**
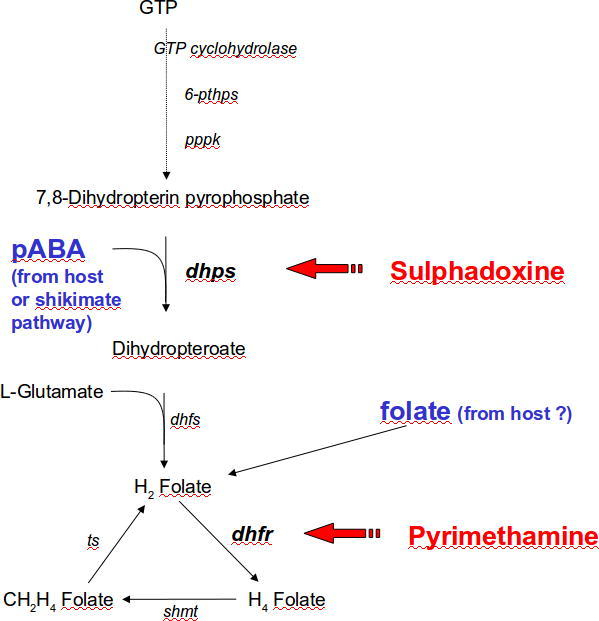
Folate pathway in *Plasmodium* parasites. Pyrimethamine (PYR) and sulphadoxine (SDX) inhibit dihydrofolate reductase (DHFR) and dihydropteroate synthase (DHPS), respectively. 4-Aminobenzoic acid (pPABA) (competitive binding with SDX) is a substrate for DHPS. Both pPABA and folate may be acquired from the host. pPABA may also be produced endogenously via the shikimate pathway. Host folate may mediate a ‘folate effect’ and hence alleviate the inhibitory action of SDX and, to a lesser extent, PYR. *6-pthps*, 6-pyruvoyltetrahydropterin synthase; *gtpch*, GTP cyclohydrolase I; *pppk*, dihydropterin pyrophosphokinase; *dhps*, dihydropteroate synthase; *dhfs*, dihydrofolate synthase; *dhfr*, dihydrofolate reductase; *shmt*, serine hydroxymethyltransferase; *ts*, thymidylate synthase.

With the evolution of chloroquine resistance in malaria parasites in Africa and southeast Asia, the combination of the antifolate drugs pyrimethamine (PYR) and sulphadoxine (SDX), commercially known as Fansidar®, was one of the mainstays of anti-malarial drug therapy. Although resistance is now widespread, it is still used in Africa, sometimes in combination with the artemisinin derivative artesunate, to treat uncomplicated malaria (Nosten and White, 2007). PYR and SDX synergistically inhibit enzyme activities of the folate biosynthesis pathway, namely dihydrofolate reductase (DHFR) and dihydropteroate synthetase (DHPS), both encoded in bifunctional enzymes by *dhfr-ts* and *pppk-dhps*, respectively (Supplementary Fig. S1). The folate pathway plays a critical role in pyrimidine and DNA synthesis. The action of antifolate drugs, the genetic basis of parasite resistance, and the complexities of the relationships between parasite genotype and in vivo and in vitro drug response phenotypes have been comprehensively investigated (reviewed in Gregson and Plowe, 2005; Hyde, 2005; Nzila, 2006).

Four point mutations in *dhfr* have been associated with the development of PYR resistance in the human malaria parasite *Plasmodium falciparum*. The S108N point mutation is crucial for the initial acquisition of PYR resistance: it interferes with the kinetics of PYR binding to DHFR (Gregson and Plowe, 2005; Hyde, 2005; Nzila, 2006). Subsequent mutations (N51I, C59R and I164L) are thought to further increase resistance (Peterson et al., 1988; Basco et al., 1995; Sirawaraporn et al., 1997; Nzila-Mounda et al., 1998). Transfection studies show that the S108N mutation reduces the response of malaria parasites to PYR (Wu et al., 1996). In populations of *P. falciparum* field isolates from Thailand, Laos and central Africa, selective sweeps have been documented around *dhfr* on chromosome 4, where genetic hitchhiking has reduced genetic diversity in PYR-resistant parasites relative to susceptible parasites (Nair et al., 2003; Nash et al., 2005; McCollum et al., 2008).

SDX is an analogue of 4-aminobenzoic acid (pABA) – a substrate of DHPS (Supplementary Fig. S1). SDX resistance has been linked with several point mutations on the *pppk-dhps* gene: S436A, A437G, K540E, A581G and A613S,T (Triglia and Cowman, 1994; Plowe et al., 1997; Triglia et al., 1997; Wang et al., 1997). A number of *dhps* allele combinations were tested in *P. falciparum* by transfection, confirming their role in SDX responses (Triglia et al., 1998). However, a number of factors have impeded a clear formal association between mutations in *dhps* and in vitro SDX and SDX/PYR (S/P) responses. For example, the SDX response phenotype may depend upon the availability of exogenous folate and pABA (Milhous et al., 1985; Krungkrai et al., 1989; Wang et al., 1997, 1999), presumably because exogenous folate ‘bypasses’ the requirement for DHPS activity (the ‘folate effect’; Wang et al., 1997), and because pABA can compete with SDX. It is also likely that other genes effect SDX (or S/P) responses. For example, the acquisition of PYR resistance by *dhfr* mutation may increase susceptibility to SDX (Hayton et al., 2002). Also, other genes may be linked to the ‘folate effect’ (Wang et al., 1997); for example, it has been proposed that mutations in the multi-drug resistance protein 1 (MRP1) may contribute to S/P resistance (Dahlström et al., 2009), possibly mediated by decreases in folate efflux from the parasite. There have been other reports of SDX or S/P resistance appearing without the apparent involvement of *dhps* mutation in both human and rodent malaria (Hayton et al., 2002; Mberu et al., 2000). This paper addresses the possible identities of such genes.

**Fig. 1:**
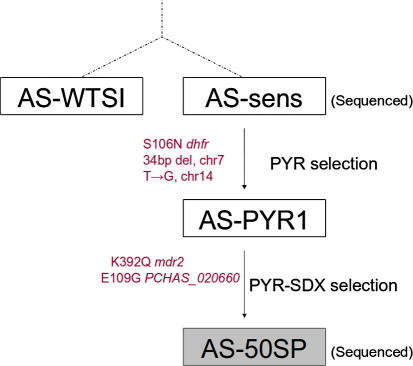
AS lineage of the *Plasmodium chabaudi* parasite clones used in the study. The lineage progenitor clone AS-sens was selected for pyrimethamine (PYR) resistance, giving rise to AS-PYR. Subsequently, AS-PYR was selected for sulphadoxine (SDX)/PYR (S/P) resistance, resulting in clone AS-50S/P with reduced susceptibility to SDX. AS-sens was also used to generate the current reference genome for *P. chabaudi*. The reference genome is termed AS-WTSI; dotted lines represent possible divergence due to passage history. Verified mutations are indicated. Dihydrofolate reductase (*dhfr*), chromosome (chr), multi-drug resistance protein 2 gene (*mdr2*).

The rodent malaria parasite *Plasmodium chabaudi* has been used as an in vivo experimental model for the generation of drug-resistant mutants by experimental evolution and the identification of the genes conferring the drug-resistance phenotypes (Carlton et al., 2001). For example, the mutation conferring PYR resistance in the experimentally selected AS-PYR clone (Fig. 1) is S106N *dhfr* (Cowman and Lew, 1990; Cheng and Saul, 1994; Culleton et al., 2005): this mutation is homologous to S108N in *P. falciparum*. As also reported in *P. falciparum* (Eastham and Rieckmann, 1983), the acquisition of PYR resistance in *P. chabaudi* is accompanied by an increase in susceptibility to SDX (Jacobs, 1964; Hayton et al., 2002). Subsequently, a single-step exposure of the AS-PYR clone to a high dose of S/P selected a mutant clone (AS-50S/P) which is more resistant to both S/P and SDX (Hayton et al., 2002) than the AS-PYR progenitor (Fig. 1). These phenotypes are genetically stable. AS-50S/P has no mutation in the *dhps* gene, nor additional mutations in the *dhfr* gene (Hayton et al., 2002). In order to map the genes underlying the modified drug response phenotypes, Quantitative Trait Locus (QTL) analysis of 16 independent cloned recombinant progeny of a genetic cross between the resistant parent AS-50S/P and the genetically distinct susceptible parent AJ suggested that loci on chromosomes 7 and 13 (chr07 and chr13) are associated with PYR, SDX and S/P responses (Hayton et al., 2002). As expected, for chr07 (containing *dhfr*), growth after PYR treatment (and S/P) was associated with the inheritance of the mutant AS allele: growth following SDX treatment was associated with inheritance of wild-type AJ alleles. For chr13 markers, growth during SDX treatment was associated with inheritance of AS alleles, whilst for PYR and S/P, growth was associated with inheritance of AJ alleles.

Those data suggested that a mutation on chr13 in AS-50S/P relative to AS-PYR increased the SDX resistance (of a PYR-resistant parasite). Here, we have re-sequenced the whole genome of the SDX-resistant clone of *P. chabaudi* (AS-50S/P) using Solexa paired-end short reads to identify point mutations, small indels (⩽3 bp) and, for key genes, copy number variations (CNVs) too.

## 2 Materials and methods

### 2.1 Parasite lines, maintenance, parasite preparation and DNA extraction

*Plasmodium chabaudi* clone AS-50S/P of the AS-lineage (Hayton et al., 2002; Fig. 1) was routinely inoculated, passaged in CBA mice (4–6 weeks) and cryopreserved as previously described (Walliker et al., 1975). Parasites were prepared and DNA extracted as previously described (Grech et al., 2002), ensuring that host white cells were removed by CF11 cellulose (Whatman, UK) and Plasmodipur filters (Eurodiagnostica, The Netherlands).

### 2.2 Illumina Solexa genome re-sequence analysis

Clone AS-sens was previously re-sequenced using the Illumina® (Solexa) platform with 36 bp single reads (Hunt et al., 2010), while clone AS-50S/P was sequenced for this study using 50 bp paired-end reads, at a mean genome coverage of approximately 45 reads per nucleotide. Individual sequence strings (reads) from each clone were aligned against an isogenic AS reference sequence (AS-WTSI (Fig. 1), provided by the Pathogen Sequencing Group, Wellcome Trust Sanger Institute, UK) using two different software packages; MAQ (Mapping and Assembly with Quality; Li et al., 2008) and SSAHA2 (Sequence Search and Alignment by Hashing Algorithm; Ning et al., 2001). A permissive insert size range (40–500 bp) was chosen for the alignment of paired-end reads. The AS-WTSI sequence data consisted of a recently completed assembly and annotation made available during this investigation (ftp://ftp.sanger.ac.uk/pub/pathogens/P_chabaudi/September_2009_assembly/). The detection of single nucleotide polymorphisms (SNPs) was performed using MAQ and SSAHA2, as described previously, with a minimum coverage of three reads chosen to call a potential SNP prior to further quality analysis (Hunt et al., 2010). Lists obtained for AS-sens and AS-50S/P were compared in order to remove candidate SNPs which did not arise within the lineage. All putative point mutations proposed by MAQ and/or SSAHA2 were verified by di-deoxy sequencing.

Small indels (⩽3 bp) were predicted using the internal algorithm of SSAHA2. The potential indel list was filtered against a list previously obtained for clone AS-sens (Hunt et al., 2010), in order to remove calls not unique to AS-50S/P. A limited number of the small indel calls were investigated by di-deoxy sequencing.

No genome-wide large indel or CNV prediction was attempted using SSAHA2 because no appropriately sequenced (i.e., using paired-end reads) AS-sens genome was available to act as a “filter” (Hunt et al., 2010) to remove the large number of candidates which are not exclusive to the mutant clone.

However, a list of potential indels (including indels >3 bp) was produced using the internal MAQ prediction algorithm. This algorithm is only available for genomes sequenced using paired-end reads. Again, no appropriate “filter” could be used to remove those indels not unique to AS-50S/P. However, the list of small indels obtained with MAQ was compared with the small indel predictions of SSAHA2. Three large indels were predicted with MAQ and these were retained as candidates.

To detect gene amplifications, the fold-coverage of sequence reads mapping to individual genes was measured using Artemis (Rutherford et al., 2000) and the coverage plot obtained from SSAHA2 (see Hunt et al., 2010). The gene-specific average fold-coverage was divided by the mean genome coverage to produce a “relative coverage” in AS-sens and AS-50S/P. A “comparative coverage” index was produced by dividing the relative coverage of the mutant clone by that of AS-sens. If the “comparative coverage” index was >1.5 it was taken as evidence of gene amplification. A known gene duplication in the multi-drug resistance gene 1 (*mdr1*, PCHAS_123820) in a clone previously selected for mefloquine resistance (AS-15MF; Cravo et al., 2003) and which has also been Solexa sequenced (unpublished data) was used as a positive control.

### 2.3 Protein function prediction

Information on predicted proteins was extracted from the PlasmoDB database (http://www.plasmodb.org/) for all mutations confirmed by di-deoxy sequencing. In the case of hypothetical proteins, the amino acid sequences were submitted to Pfam (http://www.pfam.sanger.ac.uk) and Prosite (http://www.expasy.ch/prosite/) for the identification of protein domains, functional sites and protein families. HMMTOP (http://www.enzim.hu/hmmtop/) was used for the prediction of transmembrane domains.

## 3 Results

### 3.1 Genome re-sequencing

**Supplementary Fig. S2:**
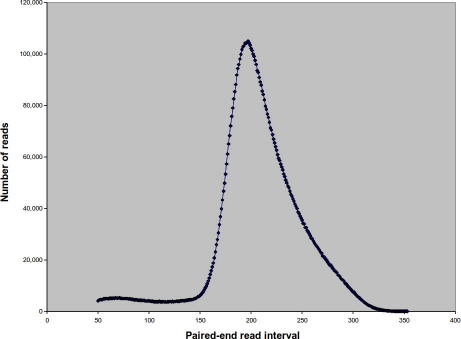
Distribution of the intervals for the paired-end reads of the *Plasmodium chabaudi* clone AS-50S/P.

The genome of clone AS-50S/P was sequenced as 50 bp paired-end reads using the Illumina short read sequencing platform (Solexa). These reads were mapped against the reference genome (assembly of September 2009, see Section 2.2) using two software packages: MAQ (Li et al., 2008) and SSAHA2 (Ning et al., 2001). In total, 19,132,804 short paired-end reads were produced. Ninety percent of those reads mapped to the genome in MAQ and 92% in SSAHA2. Of those, 17,943,606 (i.e., approximately 90% of the total reads) mapped uniquely to the reference genome in SSAHA2. The average length of the paired-end read intervals was 207 bp with the frequency distribution shown in Supplementary Fig. S2. Eighty-nine percent of the nucleotides in the genome were covered by at least 10 reads in SSAHA2. The scaffolded assembly yielded an average genome-wide read coverage of 47.0 or 44.4 reads per nucleotide (MAQ and SSAHA2, respectively).

### 3.2 SNP detection

Both MAQ and SSAHA2 were used to call SNPs. SNP calls were filtered against an equivalent list of SNPs identified by Solexa re-sequencing of the susceptible progenitor clone AS-sens (Hunt et al., 2010) in order to produce a list of predicted mutations arising in the lineage between AS-sens and AS-50S/P. Four point mutations were identified by both MAQ and SSAHA2. They appear on chr02, chr07, chr13 and chr14 (Table 1). They include non-synonymous substitutions in *dhfr* (chr07, S106N PCHAS_072830, SSAHA quality score 99), in a gene encoding an ABC transporter, multi-drug resistance protein 2 (MDR2), (chr13, K392Q PCHAS_131500, quality score 47), in a gene encoding a small conserved protein (chr02, E109G PCHAS_020660, quality score 99), and an intergenic mutation (T936,945G) lying between two genes coding for two conserved *Plasmodium* proteins (chr14, PCHAS_142590 and PCHAS_142600, quality score 40). The gene identifications (IDs) and their presumed orthologues in *P. falciparum* are given in Table 1. Those four mutations were confirmed by amplification and di-deoxy sequencing, and their origin within the AS lineage determined by sequencing AS-sens, AS-PYR1 and AS-50S/P (Fig. 1).

DHFR S106N has previously been characterised (Cowman and Lew, 1990; Cheng and Saul, 1994) and arises, as expected, in AS-PYR1 during PYR selection. The intergenic point mutation on chr14 (T936,945G) was confirmed by dideoxy sequencing as appearing first in AS-PYR1. Consultation of the PlasmoDB and PlasmoPredict databases, as well as a search for matches on the Pfam and Prosite search engines, did not yield any significant hits pointing to a potential function for the two proteins flanking this mutation.

The point mutation on chr02 on the other hand was confirmed as arising in AS-50S/P during selection by S/P. No functional predictions were made for the mutated gene (PCHAS_020660) and the E109G mutation could not be placed within any recognised protein domain.

**Supplementary Fig. S3:**
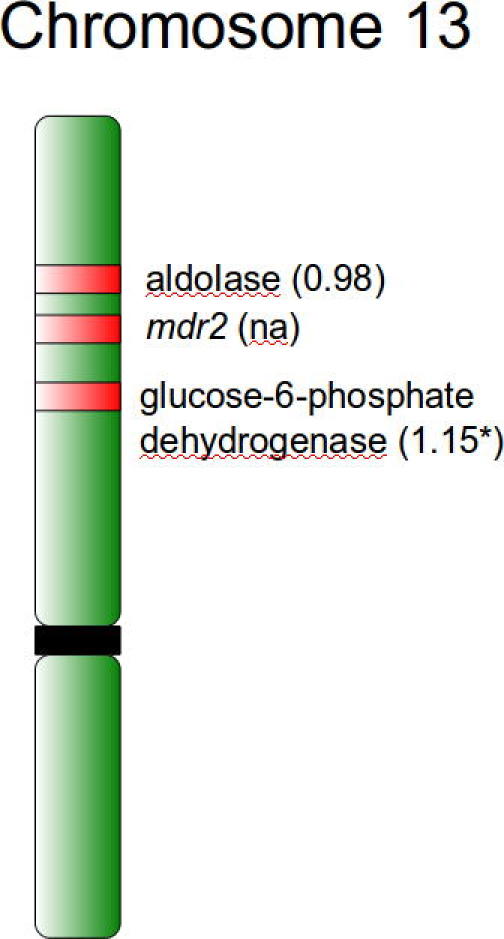
Positions of markers and genes on *Plasmodium chabaudi* chromosome 13. Multi-drug resistant protein 2 (*mdr2*), fructose-bisphosphate aldolase (*aldoI*) and glucose-6-phosphate dehydrogenase (*g6pdh*) are indicated. Numerical values next to gene acronyms indicate the level of linkage (mean difference in log parasitaemia) of the parental allele with sulphadoxine (SDX) response, as calculated by Hayton et al. (2002). The asterisk indicates that linkage is statistically significant (*P* < 0.05).

On chr13, MDR2 K392Q also arises in AS-50S/P during selection by S/P. The mutation is located within the fifth of 10 predicted transmembrane domains of this putative ABC transporter (ABCB3; Sauvage et al., 2009) protein. It substitutes a neutral polar glutamine for a positively charged lysine. Importantly, previous QTL analysis of the AS-50S/P × AJ genetic cross showed that genetic markers mapping to chr13 were linked to SDX resistance (Hayton et al., 2002). Specifically, on chr13, *mdr2* maps between those markers most closely linked to SDX resistance: it lies approximately 100 kb downstream of marker *aldoI* (currently annotated as fructose-bisphosphate aldolase, PCHAS_131180, located approximately at position 510,000) and 200 kb upstream to marker *g6pdh* (glucose-6-phosphate dehydrogenase, PCHAS_132080, located approximately at position 829,000) (Supplementary Fig. S3)*.* In order to estimate the likelihood that a potential point mutation in the proximity may have been missed due to insufficient read coverage, a region spanning 400 kb upstream and downstream of the *mdr2* gene was selected and the proportion of nucleotides within this area that were covered by less than three reads (i.e., the minimum for calling a SNP in both SSAHA2 and MAQ) was estimated as 0.4%. Across the whole of chr13, this proportion rose to 1.4%. Both values indicate a very low likelihood of any SNP having been missed on chr13 due to insufficient coverage, and strongly suggest that the mutation on *mdr2* is the sole mutation linked with the SDX-resistant phenotype.

Four additional low quality SNP calls were made by SSAHA2 only (Table 1). Excluding a putative point mutation on chr14 (T711,800G, coverage 11, quality score 37), the remaining calls were both of lower quality and lower coverage than the SNPs identified by both MAQ and SSAHA2. One call on chr14 (T951,458A) had a quality score of 10 and a read depth of 3. Both of these chr14 calls were negated by PCR and di-deoxysequencing, and therefore deemed as false-positives. The remaining two calls, namely A107,354C (read coverage of 5 and quality score of 25) and T411,999C (read coverage of 4 and quality score of 14) mapped to a composite “chromosome” of contigs not yet located to any chromosome (“bin”) and lay within or near to genes coding for members of the *chabaudi* Interspersed Repeat (*cir*) family. These two point mutations could not be verified by di-deoxy sequencing because relevant DNA fragments could not be amplified. No SNP calls were uniquely proposed by MAQ.

### 3.3 Indel detection

SSAHA2 was used to identify small indels (⩽3 bp), which were further confirmed by MAQ analysis. Potential large indels (>3 bp) were only predicted through MAQ, due to the lack of an appropriately re-sequenced (i.e., with paired-end reads) susceptible parent AS-sens to act as a filter for the SSAHA2-based analysis (see Hunt et al., 2010). A total of 62 indels were predicted (see Supplementary Table S1). Fifty-eight were classified as small indels, while four were classified as large indels. Table 1 shows the 11 indels that were either confirmed (green), rejected (red) by di-deoxy sequencing or those that were considered as being high confidence mutations (yellow), based on read coverage and SSAHA2 quality scores as described previously (Hunt et al., 2010). All large indels, with one exception (see below in this section) were classified as low confidence mutations, due to the lack of appropriate filtering in the MAQ analysis (see Section 2.2).

Only two indels (a 1 bp deletion at position 290,663 on chr10 and a 1 bp insertion at position 2,070,154 on chr14) were identified by SSAHA2 and MAQ (Table 1 and Supplementary Table S1). Di-deoxy sequencing of the deletion on chr10 indicated that this call was a false positive. Of the remaining indels, only one was analysed by di-deoxy sequencing (a single nucleotide insertion at position 854,448 on chr08) and rejected as a false positive (Table 1). No further re-sequencing was undertaken, but due to the large number of indels compared with point mutations and previous experience with a similar data set (Hunt et al., 2010) it is predicted that the vast majority of these calls will be false positives.

One indel that was not predicted by the MAQ algorithm was verified in the course of this study. Its presence was predicted based on previous whole-genome re-sequencing analysis (and validation) of related clones of the same lineage, using a comparative coverage approach based on the SSAHA2 software (Hunt et al., 2010); an approach which could not be applied here due to the lack of a paired-end sequenced AS-sens genome for comparison. The 34 bp deletion (located between positions 876,894 and 876,927 on chr7) was verified by di-deoxy sequencing (Table 1). This deletion falls immediately downstream (and 3′) of a gene encoding a conserved *Plasmodium* protein (PCHAS_072420, homologous to PF08_0067 in *P. falciparum*), which contains four transmembrane domains, a signal peptide and a ubiquitin-like fragment. It is also located upstream (and 5′) of a putative eukaryotic translation factor (PCHAS_072430, homologous to MAL8P1.83 in *P. falciparum*).

### 3.4 CNV detection

Gene copy number variation in *dhfr* and GTP cyclohydrolase have been identified (Cowman and Lew, 1990; Kidgell et al., 2006) and may modulate responses to antifolate drugs. We investigated the copy numbers of genes encoding enzymes of the folate biosynthetic pathway (Supplementary Fig. S1) using a validated analysis of the coverage of Solexa reads in these genes. Table 2 shows that the relative read-coverages for GTP cyclohydrolase I, 6-pyruvoyltetrahydropterin synthase, *dhps*, dihydrofolate synthase, *dhfr* and serine hydroxymethyltransferase were no different in AS-50S/P compared with AS-sens. In contrast, the relative read coverage for *mdr1* in AS-15MF (Cravo et al., 2003) was twice that in AS-sens, reflecting the previously known duplication in that clone.

## 4 Discussion

Of the four point mutations identified, two, namely S106N *dhfr* (chr07) and the intergenic point mutation on chr14 were already present in the PYR-resistant progenitor clone, AS-PYR1.

Two point mutations occurred between AS-PYR and the S/P-resistant clone, AS-50S/P. These are located on chr02 and chr13. There were no further mutations in *dhps* or other known folate genes which might account for the increased resistance to SDX in AS-50S/P. These data support a previous study which identifies a small number of point mutations accumulating during the evolution of other complex drug resistance phenotypes (Hunt et al., 2010) and confirms how this model and technology are well suited for complete genome re-sequencing of isogenic strains of *P. chabaudi* and identification of critical mutations conferring drug-resistance phenotypes.

Of these three mutations, that located on chr13 is most significant to SDX responses because two genetic markers most linked to SDX responses in QTL analysis mapped to chr13 (Hayton et al., 2002). These markers (numbers 19 and 37; Hayton et al., 2002) map to *g6pdh* (PCHAS_132080) and fructose-bisphosphate aldolase (PCHAS_131180), respectively (Carlton et al., 1998). The K392Q MDR2 mutation maps between these two markers (Supplementary Fig. S3), ∼216 kb upstream of the former and ∼104 kb downstream of the latter. Because no other mutations were indicated on this chromosome, we propose that the mutant AS *mdr2* allele contributes to increased resistance to SDX.

In *P. falciparum*, increased resistance to SDX in PYR-resistant parasites often involves mutations in *dhps*, but other mechanisms may exist. For example, SDX-resistant *P. falciparum* isolates with wild-type *dhps* sequences have been characterised (Mberu et al., 2000). Furthermore, recent work by Dahlström et al. (2009) has suggested that mutations (I876V and K1466R) in the multi-drug resistance protein 1 (encoded by *mrp1* (PFA0590w) may mediate an alternative mechanism modulating S/P resistance. For example, they propose that this mutation may result in higher concentrations of exogenous folate being made available for the cell. Here, we identify mutations in MDR2, another ABC transporter (Klokouzas et al., 2003; Rosenberg et al., 2006; Sauvage et al., 2009), which may function similarly.

In *P. falciparum*, MDR2 (PF14_0455) is characterised as an ABCB (pfABCB3) transporter (Sauvage et al., 2009) belonging to the same subfamily as MDR1 (PFE1150w, pfABCB1) but distinct from the MRP1 transporter (PFA0590w, PfABCC1) investigated by Dahlström et al. (2009), which belongs to the ABCC subfamily of ABC transporters. MDR2 is located both in the plasma membrane of the parasite as well as in the food vacuole membrane and contains 10 transmembrane domains and a single nucleotide binding site (Zalis et al., 1993; Rubio and Cowman, 1994). It is related to the MDR1 ABC transporter implicated in resistance to multiple anti-malarial drugs, notably mefloquine (Duraisingh and Cowman, 2005). MDR2 is associated with heavy metal ion efflux and resistance to cadmium (Rosenberg et al., 2006) but evidence of its involvement in responses to anti-malarial drugs is weak or conflicting (Ekong et al., 1993; Rubio and Cowman, 1994; Wurtz et al., 2010). MDR2 may be involved in the transport of organic anions, such as folate or pABA, as suggested for MRP1 (Dahlström et al., 2009), and due to the structural similarity between pABA and sulphadoxine, the transport of SDX too. Independently of the actual mechanism by which MDR2 mediates SDX resistance, its role in SDX-resistant *P. falciparum* strains with wild-type *dhps* sequences (Mberu et al., 2000) may now be evaluated. The role of *mdr2* mutations in generating SDX resistance might also be experimentally investigated using transfection approaches in either *P. chabaudi* or *P. falciparum*.

Previous Solexa analysis of other clones of the AS lineage of *P. chabaudi* parasites identified a 34 bp deletion on chr07 located ∼120 kb upstream of *dhfr* (Hunt et al., 2010). This mutation is present in AS-PYR1 but not AS-sens. Its relationship to PYR resistance or to possible fitness costs of a *dhfr* mutation is not known. It is located 47 bp downstream of the 3′ end of a gene (PCHAS_072420, orthologous to PF08_0067) that encodes a conserved *Plasmodium* spp. protein of unknown function. This protein is predicted to contain four transmembrane domains and a signal peptide. Previous genetic linkage data from a genetic cross between the *P. falciparum* clones Dd2 and HB3 (with different *dhfr* and *dhps* alleles) and sequence analysis of other *P. falciparum* isolates suggest the possibility that the ‘folate effect’ was associated with a gene closely linked to *dhfr* (Wang et al., 1997).

Despite the apparent simplicity of the drug/target interactions in the folate pathway, the patterns of antifolate drug susceptibility can be complex. We note that Metabolic Control Analysis (Hornberg et al., 2007) provides a conceptual framework for understanding the distributed control of flux within metabolic pathways (in contrast to the construct ‘rate-limiting step’). In folate biosynthesis, the control of pathway flux may be shared between *dhps* activity and *dhfr* activity (and other activities, such as folate transporters or GTP cyclohydrolase activity; Kidgell et al., 2006; Nair et al., 2008), and that this distribution may change as mutations in *dhps* or *dhfr* accumulate and the kinetic parameters of the enzymes change. The synergistic action of SDX and PYR, the increased susceptibility to SDX when *dhfr* mutations generate PYR resistance, the ‘folate effect’ and the role of mutations in putative folate transporters may be more clearly understood with such a theoretical framework.

To summarise, our analysis revealed several mutations in a clone of *P. chabaudi* selected for resistance by SDX–PYR drug treatment. In particular, a non-synonymous substitution on the gene encoding MDR2 was identified and found to correlate with markers previously described as being associated with SDX resistance. It is proposed that *mdr2* may underlie the SDX resistance phenotype either by directly mediating transport of the drug itself or indirectly by mediating the transport of exogenous folate (or pABA) which may counteract the activity of SDX (the so-called ‘folate effect’).

## Figures and Tables

**Table 1:** Summary table of all mutations (single nucleotide polymorphisms (SNPs) and indels) identified in the *Plasmodium chabaudi* clone AS-50S/P using the Mapping and Assembly with Quality (MAQ) and Sequence Search and Alignment by Hashing Algorithm (SSAHA2) softwares. The status of each mutation is highlighted in bold letters. “YES” indicates confirmed mutations, “high” indicates non-confirmed high confidence putative mutations (based on quality scores), “low” indicates non-confirmed low confidence putative mutations, while “NO” indicates false positives rejected after di-deoxy sequencing. Only confirmed (“YES”), rejected (“NO”) and high confidence (“high”) mutations are shown for indels and copy number variations (CNVs), whereas low confidence (expected to be negated) SNPs (“low”) are also shown. Base quality scores and indel quality scores were based on SSAHA2.

Chr’some	Type	Analysis	Start (indels only)	End	Reference base	Base in AS-50SP	Base/indel quality	Confirmation of mutation by di-deoxy sequencing	*P. chabaudi* gene ID	aa change	*P. chabaudi* nearest gene ID	*Plasmodium falciparum* orthologue
	*Putative SNPs*											
2	SNP	MAQ/SSAHA		200,295	T	C	99	YES	PCHAS_020660	E109G		PFA0250w
7	SNP	MAQ/SSAHA		994,546	G	A	99	YES	PCHAS_072830	S106 N		PFD0830w
13	SNP	MAQ/SSAHA		613,601	T	G	47	YES	PCHAS_131500	K392Q		PF14_0455
14	SNP	SSAHA		711,800	T	G	37	NO			5-PCHAS_141940	PF13_0106
14	SNP	MAQ/SSAHA		936,945	T	G	40	YES			5-PCHAS_142600	PF08_0081
14	SNP	SSAHA		951,458	T	A	10	NO			5-PCHAS_142640	PF08_0083
bin	SNP	SSAHA		107,354	A	C	25	low	PCHAS_000260			None
bin	SNP	SSAHA		411,999	T	C	14	low			PCHAS_001050-3	None

	*Putative indels*				*Size*							
2	Insert	SSAHA	378,527	378,527	1		8/12	high	PCHAS_021100			MAL7P1.154a
3	Insert	SSAHA	409,429	409,429	1		6/10	high	PCHAS_031150			PFB0560w
7	Deletion	SSAHA	779,270	779,270	1		120/46	high	PCHAS_072120			MAL8P1.65
7	Deletion	SSAHA	876,894	876,927	34			YES			PCHAS_072420-3	PF08_0067
8	Insert	SSAHA	854,448	854,448	1		2/4	NO	PCHAS_082240			PFI1065c
9	Insert	SSAHA	60,998	60,998	1		10/11	high			PCHAS_090150-5	PF11_0052
9	Deletion	SSAHA	1113,660	1113,660	1		23/24	high	PCHAS_093250			PF11_0361
10	Deletion	SSAHA/MAQ	290,663	290,663	1		26/31	NO			3-PCHAS_100680	PFD0460c
11	Insert	SSAHA	694,180	694,180	1		98/142	high			3-PCHAS_111970	PFF1025c
13	Insert	SSAHA	1703,059	1703,059	1		48/33	high			PCHAS_134540-5	MAL13P1.139a
bin	Deletion	SSAHA	262,255	262,255	1		20/18	high			5-PCHAS_000700	None

aa, Amino acid.

**Table 2:** Gene copy number by Solexa sequencing fold-coverage analysis. Comparative coverage (bold) gives an estimate of gene copy number for genes of the folate biosynthetic pathway and multi-drug resistance protein 1 (*mdr1*) in the *Plasmodium chabaudi* mutant clone AS-50S/P and wild-type clone AS-sens. Comparative coverage gives a reliable estimate of gene copy number as shown for the *mdr1* gene in mutant clone AS-15MF. *mdr1*, multi-drug resistance 1; *gtpch*, GTP cyclohydrolase I; *dhfr*, dihydrofolate reductase; *dhps*, dihydropteroate synthase; *dhfs*, dihydrofolate synthase; *6-pthps*, 6-pyruvoyltetrahydropterin synthase; *shmt,* serine hydroxymethyltransferase.

Mutant clone	Gene	Mean coverage^a^		Relative coverage^b^		Comparative coverage in mutant clone^c^
		AS-sens	Mutant clone	AS-sens	Mutant clone	
AS-50S/P	Genome^d^	39.48	44.44	1.00	1.00	**1.00**
	*mdr1*	53.39	74.59	1.35	1.68	**1.24**
	*gtpch*	45.31	34.77	1.15	0.78	**0.68**
	*dhfr*	46.01	45.48	1.17	1.02	**0.88**
	*dhps*	45.03	45.78	1.14	1.03	**0.90**
	*dhfs*	48.71	49.76	1.23	1.12	**0.91**
	*6-pthps*	55.96	54.28	1.42	1.22	**0.86**
	*shmt*	52.43	66.62	1.33	1.50	**1.13**

AS-15MF	Genome^d^	39.48	53.96	1.00	1.00	**1.00**
	*mdr1*	52.39	150.02	1.33	2.78	**2.10**

a Mean coverage is the mean number of reads for all bases within the coding region of the genome or gene.

b Relative coverage equals mean coverage of the gene divided by mean coverage for the whole genome.

c Comparative coverage is the relative coverage of the mutant clone gene divided by the relative coverage of wild-type AS-sens gene. See Section 2 for details.

d Mean read coverage across the whole genome, excluding unassigned contigs.

## Appendix A Supplementary data

Supplementary Table S1 All mutations identified uniquely in the *Plasmodium chabaudi* clone AS-50S/P following whole genome re-sequencing analysis. Column “P” provides the current status of mutations: highlighted in green are confirmed mutations, highlighted in yellow are high confidence putative mutations (based on the quality scores in column “O”), highlighted in orange are low confidence putative mutations, while red indicates false positives, i.e., putative mutations rejected after di-deoxy sequencing. This list includes lower confidence (highlighted in orange) potential indels. Read depth, base quality scores and indel quality scores were based on Sequence Search and Alignment by Hashing Algorithm (SSAHA2).
